# Detection of Protein Carbonylation in Gingival Biopsies from Periodontitis Patients with or Without Diabetes Mellitus—A Pilot Study

**DOI:** 10.3390/dj13070328

**Published:** 2025-07-18

**Authors:** Alexandra Efthymiou, Pinelopi Anastasiadou, Eleftherios Anagnostou, George Koliakos, Sotirios Kalfas, Ioannis Vouros

**Affiliations:** 1 Department of Preventive Dentistry, Periodontology and Implant Biology, School of Dentistry, Aristotle University of Thessaloniki, 54124 Thessaloniki, Greece; kalfas@dent.auth.gr; 2 Department of Oral Medicine/Pathology, Dental School, Aristotle University of Thessaloniki, 54124 Thessaloniki, Greece; apinelop@dent.auth.gr (P.A.); eleanagn@dent.auth.gr (E.A.); 3 Department of Biochemistry, School of Medicine, Aristotle University of Thessaloniki, 54124 Thessaloniki, Greece; gkoliako@auth.gr

**Keywords:** oxidative stress, protein carbonylation, diabetes, periodontal disease, anti-DNPH

## Abstract

**Background:** Protein carbonylation is an irreversible post-translational modification that is considered indicative of oxidative damage. Objective: The purpose of the study was to examine by an immunohistochemical method for the first time the extent and localization of protein carbonylation in biopsies of gingiva from periodontitis patients with or without diabetes mellitus (DM). **Methods:** These were processed for immunohistochemical staining of the carbonylated proteins, using the ENVISIOM FLEX Mini Kit, high pH, and anti-dinitrophenyl (DNP) antibody, a marker of oxidative damage to a given protein. The extent of protein carbonylation was semi-quantitatively estimated and evaluated by calculation of the Allred score (percentage of stained cells × intensity of staining). **Results:** The biopsies from periodontitis patients with diabetes mellitus (DM) exhibited higher staining scores as per the percentage of positively stained cells than the biopsies from patients with only periodontitis (means of 49.2 and 16.7, respectively), the difference being statistically significant (*p =* 0.036). The same trend was observed in the case of the combination of the above with the intensity of staining (score parameter) as well (means of 59.6 and 20.8, *p* = 0.036, respectively). **Conclusions:** An immunohistochemical method with the novelty of utilization for the first time of the anti-dinitrophenyl (DNP) antibody in gingival tissues was introduced and showed efficacy in detecting protein carbonylation indicative of oxidative stress and its impact in the pathogenesis of these two prevalent diseases of periodontitis and diabetes mellitus.

## 1. Introduction

More and more scientific evidence emerges, substantiating the role of oxidative stress in the establishment of an environment, definitely underlying, or even perhaps initiating, the pathogenesis of a variety of chronic inflammatory conditions, such as type 2 diabetes, atherosclerosis, rheumatoid arthritis, cancer, inflammatory lung disease, and periodontitis [1,2,3,4].

Periodontitis, as such, is an inflammatory condition with high prevalence, affecting 10–15% of the adult population [5], and with detrimental effects such as loss of teeth and their supporting structures, if left untreated or inadequately treated [4,6,7]. Diabetes mellitus (DM) is a chronic disorder and a common metabolic disease with a high prevalence of over 340 million people worldwide [8]. It has been termed as a state of oxidative stress, inflammation, and endothelial dysfunction [9].

Because of their similar inflammatory etiopathogenesis [10], these two serious conditions are interrelated by a bidirectional relationship, as type II diabetes (DM2) influences how severely periodontitis is expressed, and in turn, this additional inflammation may play a negative role in the disease trajectory and management of DM [11].

Oxidative stress is set to be defined as the severe imbalance between free radical production and the antioxidant counterpart, with a possible effect on tissue integrity [12]. In more detail, it is characterized by a strong battle and a resulting victory of reactive oxygen species (ROS) against the innate and adaptive immune mechanisms, which are unable to fight these precarious molecules and to initiate and orchestrate repairing processes [13].

As previously mentioned, oxidative stress is the common soil between chronic conditions, most likely as a result from systemic, low-grade inflammation [14]. Inflammatory processes, characterized by overt cytokine production, add to this stress by increasing the formation of ROS such as superoxide anions, hydrogen peroxide, and hydroxyl radicals. These, in turn, are capable of incurring damage by a variety of mechanisms, either directly or indirectly, to all biomolecules, including proteins, lipids, DNA, and carbohydrates [15,16,17], accompanied by the alteration of either their configuration and/or their properties. Basement membrane proteins have been shown to be oxidized after their exposure to ROS, affecting their interaction with cells, such as monocytes and endothelial cells [18], with this oxidation potentially even influencing the initiation of atherosclerosis.

Among a wide range of ROS-derived modifications, biomolecule carbonylation is reputable as a key figure of oxidative stress [19,20]. The generated carbonyl stress, characterized by the aggregation of reactive carbonylated species and their reactivity towards nucleophilic substrates, has deleterious effects upon physiologic processes that result in biomolecule malfunctions, increased toxicity, and even apoptotic cell death [21].

A substantial amount of evidence indicates the important role of carbonylated biomolecules in the initiation of inflammation and autoimmune responses [22,23] as a result of their malfunction. Laminins, for instance, are multidomain and multifunctional glycoproteins, key structural and functional components of the basement membranes, that contribute to many significant biological functions, such as cell adhesion, differentiation, and migration [24,25]. Concurring with the above, laminin, as well as collagen IV carbonylation, has been evidenced to strongly influence their interaction with monocytes [26,27].

In relation to the latter, Kostidou et al. [28] exhibited an increased monocyte attachment rate through the increased production of intracellular cell adhesion molecule-1 (ICAM-1) to the surface of endothelial cells. This overt monocyte attachment was associated with oxidized laminin as compared to those attached to native laminin, relating, for the first time, this monocyte impairment and dysfunctional endothelial cells to laminin oxidation. It is noteworthy to mention that an increased production of ICAM by dysfunctional endothelial cells was reported to increase, in turn, the attachment of monocytes and T lymphocytes in atherosclerosis [29].

Protein carbonylation is generally defined as an irreversible process of post-translational modification (PTM) that results in protein alteration by yielding a reactive carbonyl moiety, such as an aldehyde, ketone, or lactam [30]. It is considered specific to oxidative damage, considering the generation of free carbonyls that are not present on non-oxidized proteins [31].

**Figure 1:**
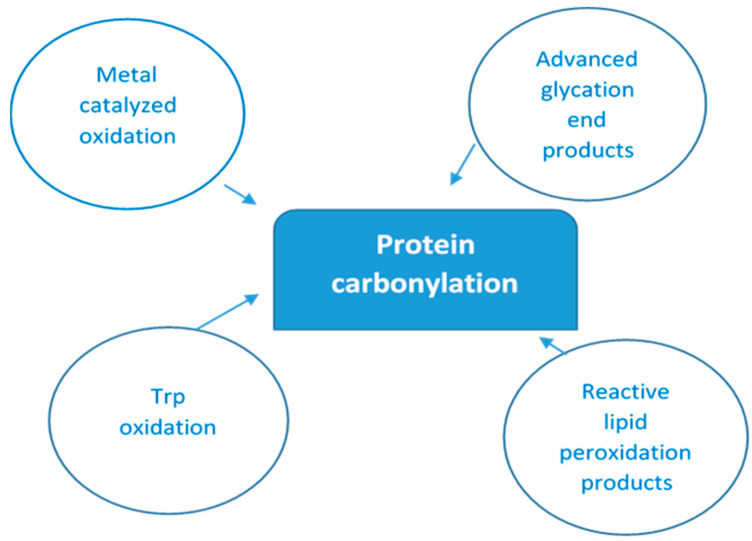
Main pathways of protein carbonylation. Protein-bound carbonyls are formed via four different mechanisms: (i) on Lys, Pro, Arg, and Thr side chains via metal-catalyzed oxidation (MCO); (ii) on Trp by direct oxidation; (iii) as adducts of advanced glycation products (AGEs) on Lys and Arg; and (iv) lipid peroxidation products of Cys, His, and Lys.

As there are so many mechanisms by which protein oxidation might be induced, along with the fact that all amino acyl side chains could be oxidatively modified, one can only speculate the numerous different types of protein oxidative modifications that can occur [32]. Figure 1 below depicts multiple ways of protein-bound carbonyls formation.

Protein carbonyl (PC) content is widely used as both a marker for oxidative stress and a measure of oxidative damage [33]. There are three categories of biochemical and analytical methods used to identify and quantify reactive carbonyls, which are (i) biochemical and immunological techniques such as immunoblotting and ELISA, providing information on the modified proteins and carbonylation levels; (ii) spectrophotometric and chromatographic assays, determining the total protein carbonyl content; and, lately, (iii) mass spectrometry (MS), identifying the modified proteins, including modification sites, and relative quantification of protein-bound carbonyls [30].

Among these, the most commonly used is the reaction of carbonyl groups with 2,4-dinitrophenylhydrazine (DNPH) to form products referred to as protein-bound 2,4-dinitrophenylhydrazones [33], which can be detected spectrophotometrically by absorbance at 370 nm or, for greater sensitivity, detected and assessed immunochemically with anti-dinitrophenyl antibodies.

On this note, it can be deduced that, despite the evident contribution of oxidative stress and consequent free radical production as a major pathogenesis mechanism in a wide range of pathologic conditions [34], as well as in normal aging, the methods to assess the extent and the way of the free radical involvement have not been validated at the same rate due to inherent difficulties in the related procedures.

Furthermore, despite numerous studies confirming the relationship between periodontitis and DM, no histological exploration has been undertaken, by now, on the role of periodontitis as an endogenous source of protein carbonyl formation and on the impact of this generated stress on the anatomy and functional properties of gingival tissues in relation to concurrent inflammation.

Hence, this study aims to investigate, for the first time histopathologically, whether inflamed periodontal tissues have the capacity to modulate and increase local oxidative stress levels in gingival tissues in individuals with or without T2DM and, even more so, whether there is an impact of this carbonylation on the basement membrane, indicating, for the first time perhaps, a novel etiopathogenesis route of gingival inflammation and ascribing to carbonylation, as a measure of oxidative damage, a more pivotal role in the initiation and remedy mechanisms.

## 2. Materials and Methods

### 2.1. Patient Characteristics

Patients were recruited during their initial, routine, or follow-up examination from the Diabetes Center of the University General Hospital of Thessaloniki AHEPA and the Departments of Preventive Dentistry, Periodontology, and implant Biology and of Dentoalveolar Surgery, Implantology, and Oral Radiology of the Dental School of Aristotle University of Thessaloniki, respectively (period of recruitment from January 2022 to June 2023).

### 2.2. Inclusion Criteria

Adults, male and female, >25 years of age, clinically healthy with no other systemic inflammatory disease aside from diabetes mellitus type Ι (DM1) or DM2 and/or periodontal disease.

### 2.3. Exclusion Criteria

Patients with acute infection in need of antibiotic treatment, cardiovascular disease, cancer, gastrointestinal disorders, skin diseases, pregnancy, lactation, arthritis, and lupus; those who had undergone periodontal treatment within the past 12 months; those who had made use of antioxidant supplements, anti-inflammatories, or antibiotics within the previous three months; and those with any other diseases of inflammatory origin.

### 2.4. Clinical Procedure

All patients enrolled (Age Range: 34–64; 9 Male and 9 Female) were scheduled for tooth extraction either due to excessive periodontal tissue loss, advanced hard tissue loss, or pericoronitis. Upon retrieval of the consent form, a complete medical history was obtained alongside a periodontal assessment (clinical and radiographical) to assess the periodontal status.

In more detail, a complete periodontal chart was obtained as follows:

➢ Pocket Probing Depth (PPD), 6 sites per tooth, utilizing a UNC-15 probe
➢ Clinical Attachment Loss (CAL), 6 sites per tooth, from CEJ to free gingival margin
➢ Gingival Bleeding Index (GI) and Plaque Index (PI), by Silness and Loe [35]

Patients were then classified accordingly into the study groups as per their diabetic and periodontal status [36], based upon their latest glycated hemoglobin (HbA1c) measurement (within the last three months) and the latest classification of periodontal diseases of 2017, according to the level of interdental clinical attachment loss, radiographic bone loss, and tooth loss, respectively [37].

### 2.5. Gingival Tissue Biopsies

Prior to extraction, biopsies of gingival tissue of 2 × 3 mm dimensions (25 in number) were obtained, initiating the incision from the top and assuring the sample contained the top of the sulcular epithelium along with connective tissue for inclusion of the basement membrane in the examined species. They were stored immediately in 10% formaldehyde solution for tissue fixation until their laboratory process within a maximum period of 2 days. In total, two investigated groups were created: one of diabetes patients with periodontitis gingival samples (14) and one of periodontitis only and non-diabetics samples (9), respectively.

A special group of two gingival biopsies of the above, termed the experimental control, derived from extractions of asymptomatic, non-infected, wisdom teeth (2/25), served as the negative control accrediting the staining technique and, thus, was not considered statistically. It is imperative to stress the latter two samples of not being incorporated into the results, and they are only mentioned in the text and table as confirmation of technique validity.

### 2.6. Immunohistochemistry {IHC} Procedure

Following sample collection and formaldehyde fixation, their processing begins as a trajectory of mechanisms encompassing several steps, the first one initiated by the application of a histokinetic (automatic tissue processing) device specially designed for the fixation, dehydration, and paraffin-embedded filtration of histological specimens of human tissue used for histological medical diagnosis by a histopathologist, followed by sectioning at a thickness of 4–7 µm (thickness dependent on type of tissue), mounting on adhesion-treated slides, and placement of the slides in a 60 °C oven overnight.

The step that ensues includes deparaffinization and rehydration using standard methods (sample protocol of slide preparation with washes of xylene, the use of graded alcohols sequentially from 96% to 80% to 70%, and rinsing in distilled water).

Antigen retrieval is the next pivotal step, with placement of the deparaffinized and rehydrated slides in retrieval buffer (high pH), utilizing the ENVISIOM FLEX Mini Kit (Dako North America Inc., Carpinteria, CA, USA) and high pH for use in immunohistochemistry, together with Dako Omnis Instruments, heating in the microwave at 100 °C for 20 min, and washing two times with ENVISION WASH BUFFER (Agilent Technologies Singapore (International) Pte Ltd., Singapore) for 5 min, with an in-between application of 200 µL of hydrogen peroxide to each slide for 20 min at room temperature.

The next pathway of the antibody application process (anti-DNPH1 rabbit polyclonal, ATLAS Company, Bee Cave State, TX, USA) involves an orchestrated sequence of the following events of application of 200 µL of diluted primary antibody to the slides, incubation at room temperature for 30 min, washing of the slides with ENVISION WASH BUFFER for 5 min, and the application of 200 μL of Envision. This phase is concluded with chromogen application (Dab Envision Chromogen, Agilent Technologies, Santa Clara, CA, USA)—specifically, the application of 200 µL of chromogen onto the slides (5–15 min, dependent on the intensity of staining)—and rinsing in distilled water.

The procedure is finalized with counterstaining, dehydration, clearing, and mounting, following a sample protocol of slide placement in hematoxylin for 15 times/dips, preparation with washes of xylene, the use of graded alcohols sequentially from 96% to 80% to 70%, and rinsing in distilled water.

### 2.7. Histopathologic Evaluation

The stained preparations were examined under a Nikon eclipse E200 LED binocular microscope (Nikon Corporation, Tokyo, Japan) (40–1000× magnification). The credibility of the aforementioned procedure was ascertained by positive and negative controls, whereby the presence and absence of cell staining served as evidence of carbonylation, respectively. Gallbladder cells were used as a positive control that verified the success of the technique, where only cells expressing the antigen showed positivity, and all other cells and structural elements were negative. For the same reason, as a negative control, where there should be no specific staining, gingival tissue (2 samples) was obtained from non-diabetic and non-periodontitis patients, as displayed in Table 1 in the experimental control group, who had their wisdom teeth extracted for other reasons [38].

Protein carbonylation was evaluated by means of the calculation of the Allred score, a current clinical practice in the research field of immunohistochemical testing and, especially, in the research field of breast cancer. It is related to its prognosis and is based on strenuous and elaborate manual counting and personal estimation of the amount and intensity of positively stained cells in immunohistochemistry (IHC)-stained slides.

Thus, the assessment of protein carbonylation is undertaken by an associate histopathologist and relies heavily on accurate inflammatory stained cell detection and classification, following immunohistochemical staining with the specific antibody (anti-DNPH) as evidence of the carbonylation process, and is based on pathologists’ manual estimation during the histological specimens’ examination [39].

**Figure 2:**
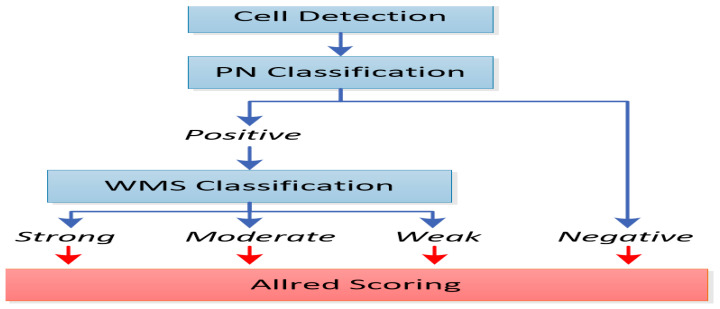
A block diagram of cell detection; positively and negatively stained (PN) cell classification; and weakly, moderately, or strongly stained (WMS) cell classification.

More specifically, as depicted in Figure 2, the Allred score represents the computation deriving from the multiplication of the percentage of positively stained cells with the appropriately categorized numerical intensity of staining. This index of intensity of staining therefore scores values from 0 to 3, which corresponds to the classification of the stained cells into negatively, weakly, moderately, and strongly stained, respectively.

Thus,

* **Allred score = percentage of stained cells × intensity of staining.**

## 3. Results

The cells that expressed immunohistochemical positivity were inflammatory cells, mainly lymphocytes and plasma cells. More specifically, the intensity was graded as 0 (negative), 1 (weak), 2 (moderate), and 3 (strong), respectively, according to the Allred scoring method. The proportion of positive cells was multiplied with the intensity grade, and the total score consequently ranged from 0 to 300. As for the basal membrane reactivity measurement, the intensity grade was multiplied with the length of the positive basal membrane. Cases scored as 0 were characterized as negative, while cases of scores between 1 and 100 were considered as mildly positive, 101–200 moderately positive, and 201–300 strongly positive. Of note, the latter category was not observed in the study.

Based on the non-parametric Mann–Whitney test applied due to abnormal data distribution, it was concluded that there was a statistically significant difference between the two test groups of patients: periodontal disease and diabetes and periodontal disease only, respectively, per the percentage of positive cells (*p*-value = 0.036) and per the score (*p*-value = 0.036), as evidenced in Table 2. More specifically, periodontal patients with diabetes exhibited higher values, on average, in comparison to periodontal patients only (means of 49.2 and 16.7, respectively). The same trend was observed in the case of the score parameter (means of 59.6 and 20.8, respectively).

Moreover, it is significant to point out that periodontitis patients presented with diabetes had the highest means of %pc and of the scores, higher also than the average means, as evident in Table 3, while, in periodontal patients without diabetes, this parameter was reduced.

**Figure 3:**
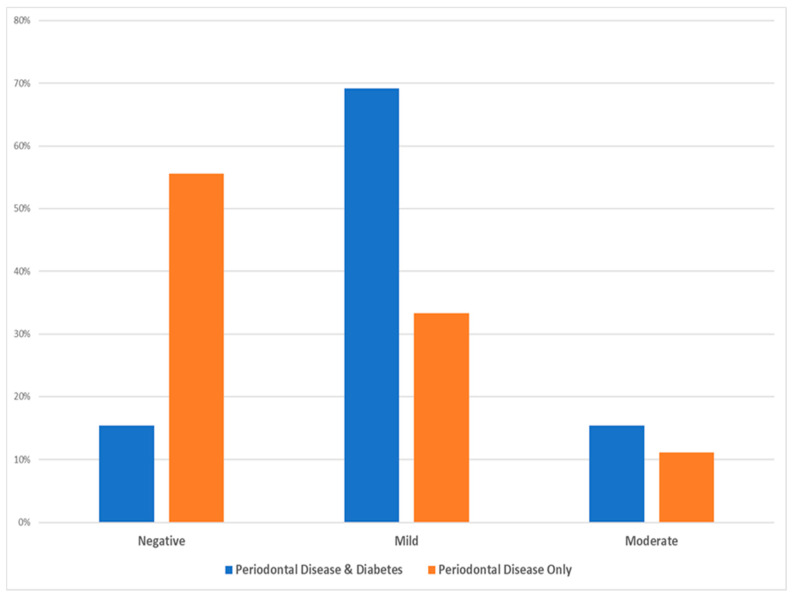
Data distribution of the two investigated groups as per the intensity of inflammatory cell staining.

Based on the statistical χ^2^ test (see Table 4), there was no statistically significant difference among the two test groups as per the intensity of cell staining (χ^2^ = 4.025, Degrees of Freedom = 2, *p*-value = 0.174, and Cramer’s *V* = 0.428), while the following Figure 3 depicts that the majority of diabetic patients with periodontal disease exhibited mild intensity and the majority of periodontal patients only showed mostly minimal (negative) intensity.

**Figure 4:**
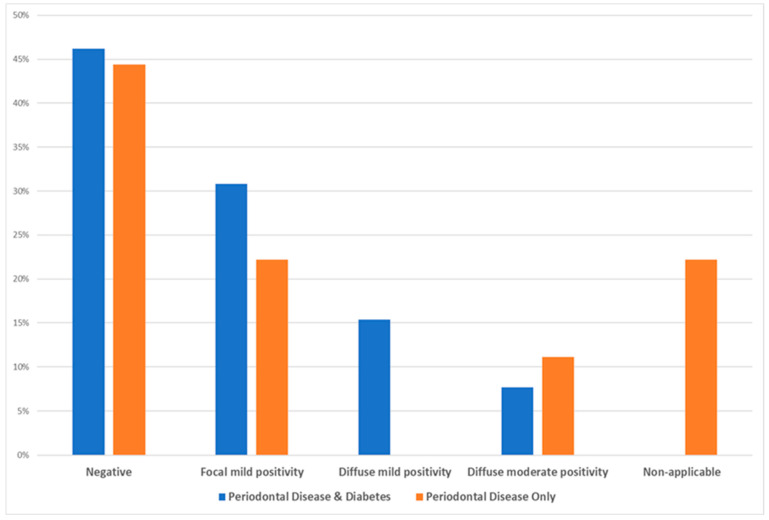
Data distribution of the two test groups as per intensity of the basal membrane staining.

Based on the statistical χ^2^ test (see Table 5), there is no statistically significant difference among the two test groups as per the intensity of basal membrane staining (χ^2^ = 4.488, Degrees of Freedom = 4, *p*-value = 0.544, and Cramer’s *V* = 0.452), while Figure 4 illustrates that the majority of samples, irrespective of the test group, showed a negative intensity for the basal membrane staining, an element quite significant with regards to the possible role and impact of carbonylation in the etiopathogenesis of periodontal disease.

### Histopathologic Evaluation

**Figure 5:**
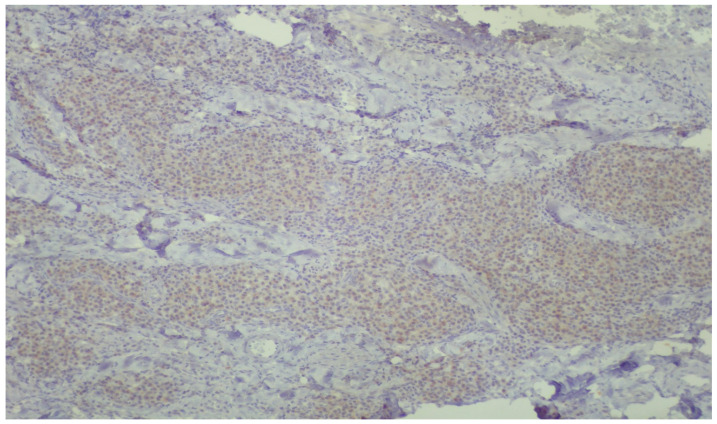
Low-power view, demonstrating diffuse, mild, positive staining of inflammatory cells for DNPH within the lamina propria (immunoperoxidase ×40).

**Figure 6:**
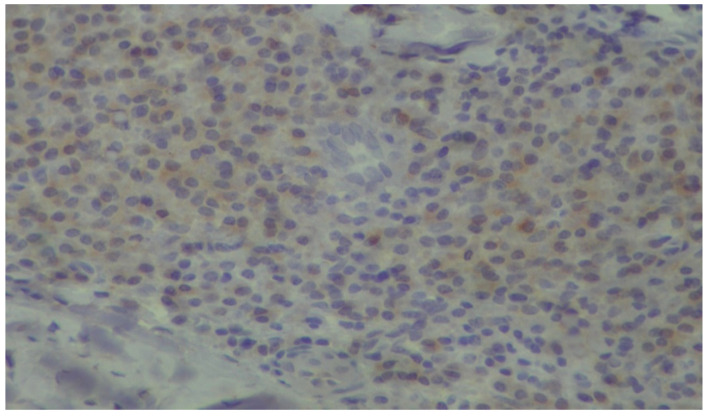
Plasma cells showing mild, cytoplasmic reactivity for DNPH (immunoperoxidase ×400).

**Figure 7:**
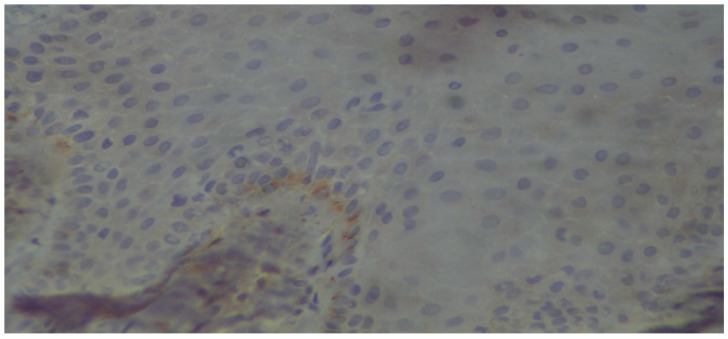
The basal membrane, as well as scattered basal cells of a stratified squamous epithelium, exhibit mild positivity (immunoperoxidase ×400).

**Figure 8:**
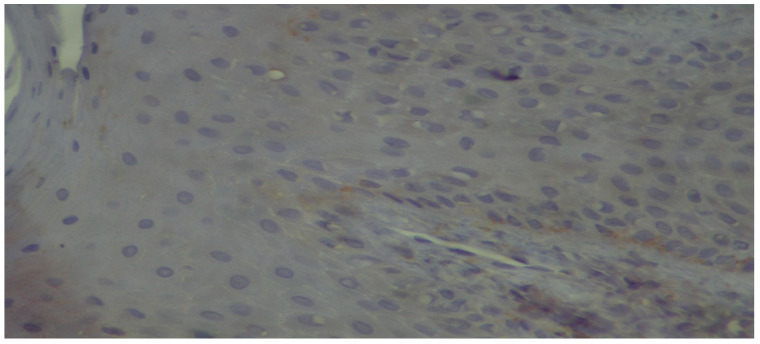
High-power view showing discontinuous positivity of the basal membrane (immunoperoxidase ×400).

**Figure 9:**
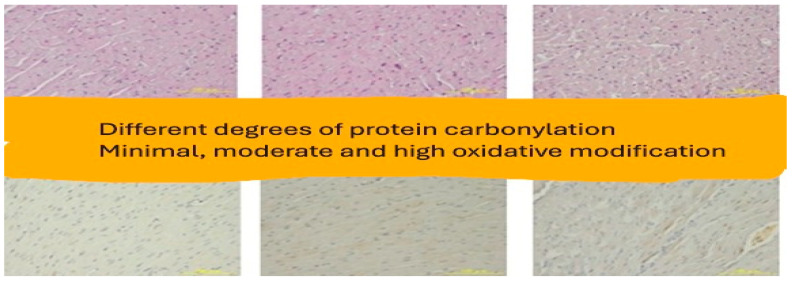
Low-power view, demonstrating varying intensities of brownish staining reflecting the amount of protein carbonyl groups (immunoperoxidase ×40).

## 4. Discussion

Periodontitis is an infection caused by bacteria, orchestrating a destructive inflammatory process due to their action and related products. It is the result of the interplay among the bacterial burden and the host’s immune capability [40].

DM2 is a chronic systemic disease related to insulin and resulting either from the pancreas’ inability to produce it enough or by the body’s inefficiency to use it effectively [41]. It has a high global prevalence, and it is reported to affect over 439 million people on the planet by the year of 2030 [42].

DM2 is considered to increase the risk for the development of periodontal diseases, whereas individuals with periodontitis have a significantly higher incidence of being diabetic when compared to patients without periodontal disease [43,44,45].

Meanwhile, oxidative stress has been shown to lead to a great degree of ‘hyperglycemia-induced tissue injury’, contributing to the early onset of diabetes mellitus and its complications [46,47]. This is supported by various studies of immunohistochemistry analysis of gingival epithelium and connective tissues, revealing the increased deposition of advanced glycation end products (AGEs) and the related activation of the receptor for advanced glycation end products (RAGEs) when periodontitis is present, as well as a 1.3-fold increase in the percentage of AGE depositions in the gingival connective tissues of patients with periodontitis and DM2 (17%) compared to non-diabetics with periodontitis (13%) [48,49,50,51].

Moreover, another interesting finding is that the percentage of AGE-positive cells in the gingival epithelium was comparable in subjects with DM2 and periodontitis (75%) compared to healthy subjects with periodontitis (70%) and without periodontitis (62.5%, *p* < 0.05), among others [52,53].

On this note, and to the best of our knowledge, immunohistochemistry analysis of the gingival epithelium and connective tissues exploring the presence and levels of carbonylated proteins in the presence of periodontitis and diabetes has not been undertaken so far. It is also the first time protein carbonylation has been assessed utilizing DNP antibody in the method, despite much attention in the last four decades in the field and the number of studies, in vitro and in vivo, using a wide panel of biochemical and analytical techniques [31,54].

Protein carbonylation is well reputed as a global marker of oxidative stress. Its significant association with various human disorders, such as Alzheimer’s disease, chronic lung disease, chronic renal failure, diabetes, sepsis, amyotrophic lateral sclerosis, cataractogenesis, cystic fibrosis, rheumatoid arthritis, and ischemia–reperfusion injury, supports its role in the pathogenesis of human disorders and also provides a strong link between disease onset/progression and oxidative stress [53,55,56,57]. Furthermore, special attention has been paid to its role in cell, tissue, and organ aging [58,59].

PC groups are relatively stable end products of protein oxidation generated by multiple forms of ROS. This justifies their wide use as biomarkers for oxidative protein damage, especially when compared to lipid peroxidation products, due to their earlier production and greater stability [60].

Their impact on and association with the periodontal status and clinical parameters, respectively, have been investigated in gingival crevicular fluid (GCF), saliva, and serum. Among the findings, it is noteworthy that higher levels of PC groups were associated with a worse periodontal status, as well as the level of PC groups being significantly correlated to the clinical periodontal parameters for periodontitis patients [61,62,63,64]. Furthermore, specific salivary proteins such as transferrin, human IgG1 heavy chain fragment, and amylase have been related to higher oxidation levels in periodontitis compared to healthy controls [65].

The basic steps in our work first included the detection of all carbonylated proteins within the specific oral region of the gums and, sequentially, along the basement membrane of gingival epithelium. This evidence was deemed crucial, as it would elucidate a new pathogenic mechanism and, possibly, novel treatment strategies alongside the histological confirmation of the bidirectional relationship between periodontitis and diabetes through the protein carbonylation route.

Our results of the immunohistochemical assessment of protein carbonylation as a measure of oxidative stress showed statistically significant differences, though minimal, among periodontitis and diabetes, as far as the levels of the carbonylated proteins were concerned, supporting even further the magnitude and interplay of oxidative stress in both assessed diseases. However, there was no significant difference among the two groups regarding the localization of the carbonylated proteins in the examined histological specimens that would enable to elucidate the possible etiopathogenesis routes of the involved conditions.

In relation to our results and in particularly the microscopic images exhibiting the staining of the carbonylation and its intensity (Figure 5, Figure 6, Figure 7 and Figure 8), it is worth mentioning at this point the relevant difficulties during the histopathology evaluation, taking into account the variation in the procedure that exists overall (Figure 9) Positively and negatively stained cells can be distinguished based on their appearance color-wise, i.e., negative cells are stained with a blue/purple hue, while positive cells are stained with a brown hue. Meanwhile, it is more challenging with respect to cell detection and the intensity of staining (WMS) classification. As far as cell detection is concerned, their close boundaries can be particularly challenging, as some cells appear too close to each other, as if they belong to single large cells, while some others have rather weak and unclear boundaries. For WMS classification, respectively, the differences between the weakly, moderately, and strongly stained cells are not always clearly obvious [39].

The subjective histopathological assessment by a histopathologist and the absence of a second evaluator or, furthermore, the lack of automated image analysis may be considered as limitations of the study. Even though the immunohistochemical methodology is robust and widely used, like any analytical and objective procedure, there exists the possibility of confounding variables that either spuriously elevate the estimates of protein carbonylation or interfere and make their histological assessment difficult [31,33,66,67,68].

Finally, the small number of gingival samples as a possible confounder in the interpretation of the findings—moreover, in a robust conclusion—cannot be overlooked. More clinical and experimental studies are needed to be able to support an alteration in our treatment remedy when considering the control of, and therapeutic protocol of, these conditions. Nevertheless, it cannot be refuted that this preliminary data could be perceived as promising and supportive with regards to further clinical research in this topic.

Recently, a series of evidence has been available that illustrated the various roles protein carbonylation can play in signal transduction processes [69,70]. These results further opened a discussion on what degree of carbonylation is required and, moreover, which specific changes in carbonyl content would have an impact and serve as cell signaling mechanisms eliciting biological responses and transducing signals [71].

However, notwithstanding the above, it is well acknowledged that protein carbonylation is related to a variety of significant pathways and mechanisms and regarded as one of the most remarkable representatives of oxidative damage [72], while PCs have been well used as protein oxidation markers.

Therefore, the carbonylation of proteins and its clinical impact appears to be a promising scientific area to continue to be explored in the future, and the novelty of the DNP antibody in the immunohistochemical method appears to have potential, with the support of additional studies, to be established as a standard or even, perhaps, state-of-the-art approach.

## 5. Conclusions

1. There is histopathological evidence for the first time of the carbonylation of proteins as a measure of oxidative damage and oxidative stress in both periodontal and diabetes diseases.
2. Periodontitis patients with DM2 exhibited higher amounts of carbonylated proteins than patients without it, displaying an additive, though minor, effect in the prevalence and possibly pathogenesis of these two diseases.
3. The introduced DNP antibody immunohistochemical method stands out to be a worthy exploratory alternative to assess protein carbonylation in gingival tissue biopsies.

## Figures and Tables

**Table 1:** Overview of the investigated groups.

Group	Gingival Samples	Diabetes Status	Periodontal Diagnosis
**Periodontal disease and diabetes** **(Test Group 1)**	13	Type II (11), Type I (2)	Periodontitis (Stages I–IV, Grades A–C)
**Periodontal disease only** **(Test Group 2)**	10	–	Periodontitis (Stages I–IV, Grades A–C)
* **Experimental control**	2	–	Non-diabetic and non-periodontitis patients

* Not included statistically. Dash (–) indicates data not applicable for non-diabetic individuals.

**Table 2:** (a) Clinical and demographic characteristics of patients with periodontal disease and diabetes. (b) Clinical and demographic characteristics of patients with periodontal disease only.

(**a**)						
**Patient ID**	**Age (yrs)**	**Sample IDs**	**HbA1c (%)**	**Diabetes Type**	**Periodontal Diagnosis (2017)**	**BoP (%)**
**Pat 1**	77	1, 2	8.4	II	Stage IV, Grade C	45
**Pat 2**	59	4, 5	6.5	I	Stage III, Grade C	30
**Pat 3**	70	6, 7, 8	6.0	II	Stage IV, Grade C	40
**Pat 4**	59	11	6.1	II	Stage IV, Grade C	50
**Pat 5**	65	3	6.3	II	Stage III, Grade C	40
**Pat 6**	55	18, 19, 22	9.1	II	Stage IV, Grade C	60
**Pat 7**	64	25	6.6	II	Stage III, Grade C	35
(**b**)						
**Patient ID**	**Age (yrs)**	**Sample IDs**	**Periodontal Diagnosis (2017)**			**BoP (%)**
**Pat 8**	65	9	Gingivitis			20
**Pat 9**	60	10	Stage II, Grade B			20
**Pat 10**	48	12, 21	Stage III, Grade B			10
**Pat 11**	58	13	Stage III, Grade B			35
**Pat 12**	55	16	Stage I, Grade B			35
**Pat 13**	43	17	Stage IV, Grade C			50
**Pat 14**	57	20	Stage II, Grade A			30
**Pat 15**	39	24	Stage I, Grade C			35
**Pat 16**	70	23	Stage IV, Grade C			45

BoP: Bleeding on Probing. HbA1c: glycated hemoglobin. Periodontal diagnosis classified according to the 2017 World Workshop on the Classification of Periodontal and Peri-Implant Diseases and Conditions. Sample IDs refer to individual gingival tissue samples collected per subject.

**Table 3:** Descriptive statistics of the parameters of the Allred scores of protein carbonylation.

Statistical Results				
Parameter	Patient Group	Sample Count	Mean ± SD	*p*-Value
**% of Positive Inflammatory Cells**	Periodontal Disease and Diabetes (Test)	13	49.2 ± 35.97	0.036
	Periodontal Disease Only (Control)	9	19.7 ± 34.40	
**Allred Score (% Positive Cells × Intensity)**	Periodontal Disease and Diabetes (Test)	13	59.6 ± 49.52	0.036
	Periodontal Disease Only (Control)	9	20.8 ± 34.21	

Data are presented as the **mean ± standard deviation (SD)**. The Allred score is calculated by multiplying the percentage of positively stained inflammatory cells by the intensity of staining. Staining intensity was scored as follows: 0 = negative, 1 = mild, 2 = moderate, and 3 = intense.

**Table 4:** Data distribution of the immunohistochemical (IHC) staining intensity of the inflammatory cells.

Staining Intensity	Periodontal Disease and Diabetes (Test Group)	Periodontal Disease Only (Control Group)	*p*-Value
**Negative (0)**	2 (15.4%)	5 (55.6%)	
**Mild (1)**	9 (69.2%)	3 (33.3%)	
**Moderate (2)**	2 (15.4%)	1 (11.1%)	
**Chi-square test**			0.174

The HC staining intensity was scored as 0 = negative, 1 = mild, and 2 = moderate. No statistically significant difference was found between groups (χ^2^ = 4.025, df = 2, *p* = 0.174, and Cramér’s V = 0.428).

**Table 5:** Distribution of the IHC staining intensity in the basal membrane.

Staining Pattern	Periodontal Disease and Diabetes (Test Group)	Periodontal Disease Only (Control Group)	*p*-Value
**Negative**	9 (69.2%)	7 (77.8%)	
**Focal Mild Positivity**	2 (15.4%)	1 (11.1%)	
**Diffuse Mild**	1 (7.7%)	1 (11.1%)	
**Diffuse Moderate**	1 (7.7%)	0 (0.0%)	
**Non-applicable/Missing**	0 (0.0%)	0 (0.0%)	
**Chi-square test**			**0.544**

IHC staining intensity of the basal membrane was categorized as negative, focal mild positivity, diffuse mild, and diffuse moderate. No statistically significant difference was observed between the test and control groups (χ^2^ = 4.488, df = 4, *p* = 0.544, and Cramér’s V = 0.452).

## Data Availability

The original contributions presented in this study are included in the article. Further inquiries can be directed to the corresponding author(s).
